# A Brief Questionnaire to Assess Post-Exertional Malaise

**DOI:** 10.3390/diagnostics8030066

**Published:** 2018-09-11

**Authors:** Joseph Cotler, Carly Holtzman, Catherine Dudun, Leonard A. Jason

**Affiliations:** Center for Community Research, Department of Psychology, DePaul University, Chicago, IL 60604, USA; Jcotler@depaul.edu (J.C.); CholtzM1@depaul.edu (C.H.); Cdudun@depaul.edu (C.D.)

**Keywords:** post-exertional malaise, chronic fatigue syndrome, myalgic encephalomyelitis

## Abstract

Post-exertional malaise (PEM) is a key symptom of myalgic encephalomyelitis (ME) and chronic fatigue syndrome (CFS). Currently, five PEM-items from the DePaul Symptom Questionnaire (DSQ) were recommended as a first step in measuring this symptom for patients with ME and CFS by the National Institutes of Health/Centers for Disease Control and Prevention (NIH/CDC) Common Data Elements’ (CDE) working group. The second step in this process, as recommended by the NIH/CDC CDE working group, involves assembling information from various sources to confirm the presence of PEM. There have not been any efforts, to date, to standardize this second-step process in the assessment of PEM. The current study examined whether five supplementary items on the DSQ could be used to operationalize the second step of the recommendations made by the NIH/CDC CDE working group. The five supplementary DSQ PEM duration items correctly categorized patients with ME or CFS 81.7% of the time, while incorrectly categorizing multiple sclerosis (MS) and post-polio syndrome (PPS) as ME or CFS only 16.6% of the time. The findings suggested that a PEM second-step process could be operationalized using supplementary DSQ items.

## 1. Introduction

Recently, the National Institutes of Health/Centers for Disease Control and Prevention [1] Common Data Elements’ (CDE) working groups were involved in an effort to specify instruments and methods for assessing the different domains of myalgic encephalomyelitis (ME) and chronic fatigue syndrome (CFS). One of its working groups focused on post-exertional malaise (PEM), a core symptom of ME and CFS. PEM involves an abnormal response (e.g., an inappropriate loss of physical and mental stamina, rapid muscular and cognitive fatigability) following physical, cognitive, emotional, or orthostatic exertion [2,3,4]. The NIH/CDC CDE’s PEM working group recommended that PEM be assessed using a two-step process, with the first step involving five PEM items from the DePaul Symptom Questionnaire (DSQ) [5], and then a second step in which the clinician evaluates these responses in light of other information. Examples of other information could include other questions on the DSQ, the clinician’s own evaluation, previous medical records, other patient-reported scales, etc., to make a final decision regarding the assessment of PEM.

Given that the DSQ’s five PEM items were included in this NIH/CDC CDE’s PEM working group recommendation, the authors provide information below regarding the derivation of these PEM items as well as issues regarding its reliability. Jason et al. [6] initially developed a scale measuring five dimensions that were used to distinguish patients with ME and CFS from controls. Using a factor analysis, the five DSQ PEM items that the NIH/CDC CDE’s working group recommended as a first step emerged as one factor. This PEM factor had good internal reliability as well as good test-retest reliability [7]. Subsequently these items were included in the DePaul Symptom Questionnaire (DSQ) [8], and factor analyses of this questionnaire have consistently found a PEM factor score [7,9]. In another study, using these five PEM DSQ items, 97% of the sample (*n* = 704) of patients with ME and CFS indicated that they experienced at least one of these PEM symptoms at least half the time and of a moderate or greater severity [10]. These five PEM DSQ items, therefore, provide an efficient and reliable screening mechanism for NIH/CDC CDE’s recommended first stage to identify PEM in patients with ME and CFS.

The NIH/CDC CDE’s PEM working group recommended that these first-step DSQ PEM items should be supplemented in a two-step process involving a clinician evaluation that included consideration of other information, such as whether responses were related to overwork or to exercise avoidance. It is possible that supplemental items on the DSQ could be used for this purpose. As an example, an additional set of PEM-related items from the DSQ assesses the duration of the PEM symptoms, and duration has been specified in ME and CFS case definitions [2,3,4,11]. Several studies have indicated that the duration of symptoms is possibly a unique aspect of PEM among patients with ME and CFS. For example, Jason et al. [12] found that 75% of participants experienced PEM exacerbation more than 24 h after engaging in light activity, a finding comparable to that found by others [13].

The aim of the current study was to examine how the utilization of supplemental DSQ PEM items may serve to operationalize the second step in the assessment of PEM. The authors’ hypothesis was that a group of supplementary DSQ PEM questions, which were not part of what had been recommended by the NIH/CDC CDE’s PEM working group, might be effective in the second-step PEM evaluation process, and thus help clinicians identify PEM.

## 2. Materials and Methods

### 2.1. Participants

Approval was obtained from the DePaul University Institutional Review Board (PF020317PSY) to conduct this study through 02/02/2019. Individuals were recruited through email requests to national foundations and posts to support groups, research forums, and social media outlets. Participants completed an online informed consent process and subsequently responded to study measures on REDCap [14], a web-based survey creation tool. Individuals were not required to complete all questionnaire items at one time; they were able to save their responses and return to continue the survey as often as needed. Diagnoses were obtained from the online questionnaire through participant self-reports. The current study included those with myalgic encephalomyelitis and/or chronic fatigue syndrome (*n* = 376), multiple sclerosis (MS) (*n* = 157) or post-polio syndrome (PPS) (*n* = 167). Those with comorbid diagnoses of ME and/or CFS with MS or PPS were excluded from the analyses.

The sample was 82.3% female and 14.6% male. They were predominantly White/Caucasian (95.4%) with 1.0% identifying as Asian, 0.7% identifying as Black, 0.7% identifying as American Indian, and 2.1% identifying as “Other”. The age range of the sample was between 18 and 86 years old (*M* = 53.02, *SD* = 15.00).

### 2.2. Measures

#### 2.2.1. First-Step DSQ Items

Participants completed the DSQ which assessed the frequency and severity of ME and CFS symptoms [8]. The following five DSQ PEM items were recommended as a first step by the NIH/CDC CDE’s working group: “A dead, heavy feeling after exercise”, “Muscle weakness even after resting”, “Next day soreness after everyday activities”, “Mentally tired after the slightest effort”, and “Physically drained after mild activity”. These five DSQ PEM items were designed to assess the frequency and severity of PEM over a six-month time frame. Participants rated each PEM symptom’s frequency for the past six months on a 5-point Likert scale: 0 = *none of the time*, 1 = *a little of the time*, 2 = *about half the time*, 3 = *most of the time*, and 4 = *all of the time*. Similarly, participants rated each symptom’s severity over the past six months on a 5-point Likert scale: 0 = *symptom not present*, 1 = *mild*, 2 = *moderate*, 3 = *severe*, 4 = *very severe*. The DSQ has been shown to have good test-retest reliability [7], and the five DSQ PEM items have good internal reliability (α = 0.84).

#### 2.2.2. Supplemental DSQ PEM Items

None of the supplemental DSQ PEM questions were rated for frequency or severity, as occurred for the items referred to above that comprised the PEM factor used in the NIH/CDC CDE’s first-step process.

*PEM Duration.* Three additional PEM items within the DSQ examined duration of symptom exacerbation after activity. Participants were initially asked two questions: “Do you experience a worsening of your fatigue/energy related illness after engaging in minimal physical effort” and “Do you experience a worsening of your fatigue/energy related illness after engaging in mental effort”. If participants answered ‘yes’ to either of these questions, they were then presented with a question measuring PEM duration: “If you feel worse after activities, how long does this last”. Participant responses of PEM duration were coded as: 0 = *Not having a problem with energy/fatigue*, 1 = *1 h or less*, 2 = *2–3 h*, 3 = *4–10 h*, 4 = *11–13 h*, 5 = *14–23 h*, and 6 = *24 h or more*. Both branching logic questions of symptom exacerbation due to physical activity (*k* = 0.84) and symptom exacerbation due to mental activity (*k* = 0.74) have good test-retest reliability [7]. 

*Quick Recovery.* The fourth supplementary PEM item assessed how quickly patients would recover from activities that typically occurred with healthy individuals. The NIH/CDC CDE’s PEM working group recognized that the DSQ 5-item first step had some limitations, particularly in terms of the breadth of the symptoms covered, and in its ability to detect PEM triggered by stressors other than physical activity. The next supplemental DSQ question might deal with these concerns, as participants were asked “If you were to become exhausted after actively participating in extracurricular activities, sports, or outings with friends, would you recover within an hour or two after the activity ended?” This item was previously demonstrated as having good test-retest reliability (*k* = 0.88) [7].

*Exercise Exacerbation.* Another limitation indicated by the NIH/CDC CDE PEM’s working group was that patients who already modified their activities to avoid or reduce PEM may potentially show up as false negatives on the PEM subscale. The fifth supplementary PEM item dealt with this concern by evaluating whether participants were not exercising because it made their symptoms worse. Participants were asked “If you do not exercise, is it because exercise makes your symptoms worse?” This item was previously demonstrated as having good test-retest reliability (*k* = 0.79) [7].

### 2.3. Statistics

Data mining techniques identify which items best predict class membership and are useful for diagnosis and assessment. In this study, decision trees were used to analyze the symptom data and determine which symptoms were distinctly associated with ME and CFS, compared to MS or PPS. For this analysis, symptom scores were calculated by combining the scores of frequency and severity into a score on a 100-point scale. The resulting variables were then put into a decision tree analysis.

Decision trees consist of a series of binary choices (or branches) that end with a classification of participants. At each branch the computer decides which symptom would best predict classifications, in this case whether a participant has ME and CFS, MS, or PPS, with the ultimate goal of distinguishing ME and CFS from MS and PPS. The process continues and more symptoms continue to separate the three groups until the tree reaches a balance between classification accuracy and generalizability to new data. The decision trees were created in SPSS statistic software using the Classification and Regression Tree (CRT) algorithm. Because of the unbalanced samples, the authors created a random subsample (*n* = 125) of each group prior to running each tree. This meant that each tree captured 80% of the distribution of participants with MS, 75% of the distribution of participants with PPS, and 33% of the distribution of participants with ME and CFS. While the samples were disproportionate, the number of responses from both groups was large enough to produce reliable results. They then ran 100 trees with the five supplemental DSQ PEM items. They examined the number of times the additional PEM symptoms appeared in the decision trees. If a symptom appeared in the first tier, it was the most important in that tree for separating the patients with ME and CFS from those with MS or PPS. Those that appeared in tier 2 were the second most important in the tree. The level of importance in separating the samples into the two distinct groups lessened with each level. It was noteworthy if a symptom appeared multiple times. In that case, the aim was to assess how well the trees classified ME and CFS responses from MS or PPS responses. Sensitivity assessed how well the trees identified those who had ME or CFS correctly. Specificity assessed how well the trees discriminated patients with MS and PPS from patients with ME and CFS.

## 3. Results

Five supplemental DSQ PEM items were incorporated into the decision tree analyses to evaluate sensitivity and specificity among the three chronic illnesses. Table 1 presents the additional PEM dimension symptoms in order of the frequency with which they differentiated ME and CFS from MS and PPS. Symptoms appearing in tier 1 differentiated the greatest number of patients, followed by tier 2, tier 3, and tier 4. For the supplemental DSQ PEM items, the average sensitivity and 1-specificity results of the decision trees were calculated. The supplementary DSQ PEM items correctly categorized patients with ME or CFS 81.7% of the time, while incorrectly categorizing patients with MS and PPS as patients with ME or CFS only 16.6% of the time. Table 2 and Table 3 show the percentage of participants who reported having the five supplemental DSQ PEM symptoms by their indicated illness. Patients with ME and CFS were slower to recover from exertion than patients with MS or PPS [χ^2^ (2, *n* = 697) = 194.49, *p* < 0.001], and more likely to experience PEM through mental exacerbation [χ^2^ (2, *n* = 699) = 169.69, *p* < 0.001] (see Table 2). Additionally, a greater proportion of patients with ME and CFS experienced PEM lasting 24 h or more compared to those with MS and PPS [χ^2^ (2, *n* = 700) = 350.54, *p* < 0.001] (see Table 3).

### Receiver Operating Characteristic

**Figure 1:**
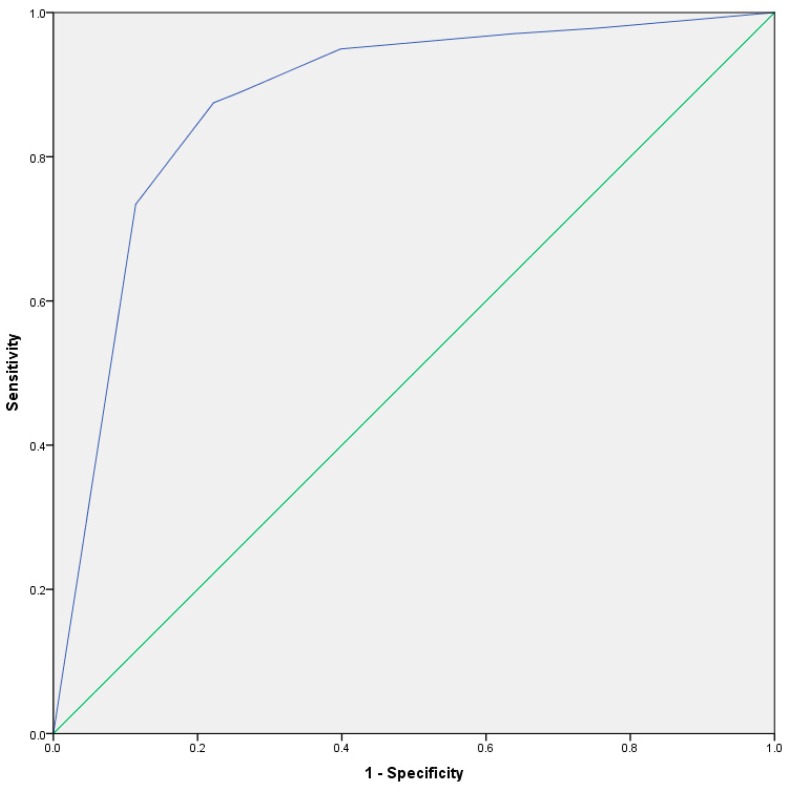
Receiver operating characteristic (ROC) curve for PEM duration.

As PEM duration was prominent in the decision tree as indicated in Table 1, further receiver operating characteristic (ROC) analysis compared whether this item could differentiate participants with ME and CFS from MS and PPS (see Figure 1). PEM duration had an area under the curve (AUC) of 0.88 (*SE* = 0.1, *CI* = 0.85–0.90, *p* < 0.001). A previous study [13] indicated that most patients with ME and CFS experience PEM lasting longer than 12 h, whereas some case definitions require lasting more than 24 h [11]. Therefore, the authors examined PEM duration from 14 to 23 h and 24 h or more. For those whose symptoms lasting 14–23 h, Sensitivity = 87.5% and Specificity = 77.8% whereas for those with symptoms lasting 24 h or more, Sensitivity = 73.4% and Specificity = 88.6%. Patients who report that their PEM lasts 14 to 23 h, had a ME or CFS diagnosis 77.8% of the time. This increased to 88.6% if patients report that their PEM lasts 24 h or more. On the other hand, patients who reported that their PEM lasts for less than 14 h had a MS or PPS diagnosis 84.2% of the time.

## 4. Discussion

By incorporating supplemental DSQ items assessing PEM in the second step of the NIH/CDC CDE’s working group recommendations, false positives were reduced to 16.60%, while the sensitivity remains above 80% when comparing ME and CFS to MS and PPS. The results demonstrate the utility of the supplemental PEM-based DSQ items as a second step in the screening process.

Of the five supplemental items, the PEM duration DSQ item was the most effective in differentiating ME and CFS from MS and PPS. Many patients with MS are able to engage in exercise without experiencing symptom exacerbation [15]. Similarly, many patients with PPS report an improvement of fatigue and other physical symptoms after engaging in exercise [16]. Therefore, PEM duration, in which exertion has prolonged effects, occurs more often and with greater duration among those with ME and CFS relative to MS and PPS. Because these additional DSQ PEM duration items can help differentiate ME and CFS from those with at least two other chronic conditions, these items might have appeal to clinicians who often have limited periods of time to make these types of diagnostic decisions [17]. PEM duration criterion as lasting 14–23 h after exertion, or 24 h or more after exertion, differentiates those with ME and CFS from MS and PPS (see Table 3).

Results for the two time periods did vary and setting the criterion at PEM duration of 14 or more hours (including responses of both ‘14–23 h’ and ‘24 h or more’) would include the majority (87.5%) of patients with ME and CFS in the screening process, while discriminating out a large number (77.8%) of patients with other chronic illnesses. Setting the criterion at 24 h or more would include a smaller subset of patients with ME and CFS (70.3%) but exclude a greater proportion of patients with other chronic illnesses (88.6%). Using the robust criterion of 14–23 h could be thought of as a clinical criterion, which is broader than a research criterion that could be 24 h or more. Further research is needed to assess whether those in these two time frames actually are different in terms of levels of disability or reductions in functioning.

One limitation of this study was that both participant diagnoses and the assessment of PEM were based on participant self-reports. Obtaining in-person physician diagnoses would have strengthened this study. In addition, some consider ME and CFS two distinct illness [18], whereas others have suggested that it might be difficult to separate these two illnesses [19,20,21]. If there are two illnesses, then collapsing groups would complicate interpretations of the results. It is beyond the scope of this article, but there might be a solution to this diagnostic issue if a more broad clinical case definition was used that could be referred to as CFS (using the Fukuda et al. criteria). Alternatively, the Canadian Consensus Criteria could be used for more research purposes, referred to as ME.

With regard to the self-report PEM measure, there are other methods for examining exertion and PEM such as cardiopulmonary exercise testing (e.g., VO_2_ peak, heart rate, and minute ventilation) [22]. While this method has high validity and reliability [23], cardiopulmonary exercise testing for patients with ME and CFS is not covered by insurance [24], and standard testing ranges from $165 to $350, while more complex cardiopulmonary exercise tests can cost up to $1605 [25]. Therefore, self-reporting data has certain advantages in terms of expense to patients with ME and CFS due not only to costs, but also to the time constraints placed on physicians to make an expedient diagnosis [17]. However, if patients with ME and CFS have greater access to medical care due to a lower financial entry barrier, then the choice of metric is in favor of the Cardiopulmonary Exercise Stress Test (CPET). Future studies should explore the relationship of the DSQ PEM items to cardiopulmonary exercise testing.

In summary, this article suggests that the two-step process recommended by the NIH/CDC CDE’s working group can be further operationalized using supplemental DSQ PEM items. As has been found in prior data sets, the first step involving five DSQ PEM items identifies the vast majority of patients (97%) in Jason et al. [10] and 99% in the current sample. Using the supplementary five DSQ PEM items, and in particular the duration of symptoms, those with ME and CFS can be differentiated from those with at least two other fatiguing illnesses, MS and PPS. Further research is needed to determine whether the supplementary DSQ PEM items can also be successful in differentiating those with ME and CFS from other chronic illnesses. While the 10 DSQ PEM items could be considered a subscale of the DSQ, what the authors will now call the DSQ-PEM (see Appendix A), these 10 items were derived from a more general instrument (DSQ) that assesses multiple symptoms of ME and CFS (see Appendix B for scoring rules regarding DSQ-PEM). An important next step, recommended by the NIH/CDC CDE PEM’s working group, is to create an instrument with the sole purpose of more comprehensively assessing PEM. In addition, there is a need to provide clear scoring rules and thresholds for all symptoms of ME and CFS [26].

## Figures and Tables

**Table 1:** Number of times all symptoms appeared in decision trees.

Symptom	Tier 1	Tier 2	Tier 3	Tier 4
PEM duration	100	2	10	0
Mental Exacerbation	0	38	17	0
Exercise Exacerbation	0	19	0	0
Quick Recovery	0	0	0	2
Physical Exacerbation	0	0	0	0

**Table 2:** Percentage of supplemental DePaul Symptom Questionnaire (DSQ) post-exertional malaise (PEM) questions reported positive by patient illness.

Symptom	MS % (*n*)	ME and CFS % (*n*)	PPS % (*n*)
Quick Recovery	42.3 (66)	1.3 (5)	38.3 (64)
Exercise Exacerbation	10.8 (17)	47.6 (179)	34.1 (57)
Physical Exacerbation	65.6 (103)	94.7 (356)	68.3 (114)
Mental Exacerbation	55.4 (87)	91.4 (342)	34.9 (58)

**Table 3:** PEM duration reported by patient illness.

Symptom	MS % (*n*)	ME and CFS % (*n*)	PPS % (*n*)
No Exacerbation	21.0 (33)	2.1 (8)	12.3 (86)
≤1 h	10.8 (17)	0.8 (3)	13.2 (22)
2–3 h	28.7 (45)	2.1 (8)	19.8 (33)
4–10 h	17.2 (27)	6.1 (23)	11.4 (19)
11–13 h	3.2 (5)	1.3 (5)	3.6 (6)
14–23 h	10.2 (16)	14.1 (53)	11.4 (19)
≥24 h	8.9 (14)	73.4 (276)	13.8 (23)

Receiver Operating Characteristic

## Appendix A

For each symptom below, please circle one number for frequency and one number for severity:

Please complete the chart from left to right.

**Table:**

Symptoms	Frequency: Throughout the **past 6 months**, **how often** have you had this symptom? For each symptom listed below, circle a number from: **0 = none of the time** **1 = a little of the time** **2 = about half the time** **3 = most of the time** **4 = all of the time**	Severity: Throughout the **past 6 months**, **how much** has this symptom bothered you? For each symptom listed below, circle a number from: **0 = symptom not present** **1 = mild** **2 = moderate** **3 = severe** **4= very severe**
1. Dead, heavy feeling after starting to exercise	0 1 2 3 4	0 1 2 3 4
2. Next day soreness or fatigue after non-strenuous, everyday activities	0 1 2 3 4	0 1 2 3 4
3. Mentally tired after the slightest effort	0 1 2 3 4	0 1 2 3 4
4. Minimum exercise makes you physically tired	0 1 2 3 4	0 1 2 3 4
5. Physically drained or sick after mild activity	0 1 2 3 4	0 1 2 3 4

For each question below, choose the answer which best describes your PEM symptoms.

**Table:**

6. If you were to become exhausted after actively participating in extracurricular activities, sports, or outings with friends, would you recover within an hour or two after the activity ended?	Yes			No		
7. Do you experience a worsening of your **fatigue/energy related illness** after engaging in minimal physical effort?	Yes			No		
8. Do you experience a worsening of your **fatigue/energy related illness** after engaging in mental effort?	Yes			No		
9. If you feel worse after activities, how long does this last?	<1 h	2–3 h	4–10 h	11–13 h	14–23 h	≥ 24 h
10. If you do not exercise, is it because exercise makes your symptoms worse?	Yes			No		

## Appendix B

DSQ-PEM Scoring
Scoring Step 1
Items 1–5: A frequency and severity score of 2, 2 on any items 1–5 is indicative of PEM.
Scoring Step 2
Items 7, 8: Either item 7 or 8 must have an answer of yes to indicate an ME and/or CFS dx.
Item 9: A response of >14 h is needed to indicate an ME and/or CFS dx.
Items 6, 10: Neither item indicates an ME and/or CFS diagnosis, but provides a description of patient PEM for clinical evaluations.
